# Scoring functions for transcription factor binding site prediction

**DOI:** 10.1186/1471-2105-6-84

**Published:** 2005-04-04

**Authors:** Markus Friberg, Peter von Rohr, Gaston Gonnet

**Affiliations:** 1 Institute of Computational Science, ETH, 8092 Zurich, Switzerland

## Abstract

**Background:**

Transcription factor binding site (TFBS) prediction is a difficult problem, which requires a good scoring function to discriminate between real binding sites and background noise. Many scoring functions have been proposed in the literature, but it is difficult to assess their relative performance, because they are implemented in different software tools using different search methods and different TFBS representations.

**Results:**

Here we compare how several scoring functions perform on both real and semi-simulated data sets in a common test environment. We have also developed two new scoring functions and included them in the comparison. The data sets are from the yeast (*S. cerevisiae*) genome.

Our new scoring function LLBG (least likely under the background model) performs best in this study. It achieves the best average rank for the correct motifs. Scoring functions based on positional bias performed quite poorly in this study.

**Conclusion:**

LLBG may provide an interesting alternative to current scoring functions for TFBS prediction.

## Background

The TFBS prediction problem can be defined as follows: Given *N *hypothetically co-regulated genes and their promoter sequences *S *= {*S*_1_, *S*_2_, ..., *S*_*N*_} (typically 1000 bp upstream of each gene, although they can be much longer in higher eukaryotes), search for motifs that are overrepresented in *S *compared to the set *A *of all promoter sequences in the genome. Ideally, the most overrepresented motif is the TFBS. A recent review on both biological and computational aspects of TFBS prediction is [1]. Another review focusing more on the computational aspects is also available [2].

Many software tools exist for TFBS prediction, e.g. Consensus [3], MEME [4,5], AlignACE [6], BioProspector [7], and MDscan [8]. These tools can be classified according to three criteria:

1. TFBS representation: How a putative TFBS is represented, e.g. consensus sequence [9,10], PSFM (position specific frequency matrix) [7], Bayesian network [11] and HMM [12].

2. Search method: How promoter sequences are searched for putative TFBSs, e.g. greedy search [3], Gibbs sampling [13] and deterministic iterative search [8].

3. Scoring function: How a newly found PSFM (or any other TFBS representation) is scored to distinguish real binding sites from background noise.

In this paper we will focus on the scoring function, which is a crucial part of any TFBS prediction software. Many scoring functions for TFBS prediction have been proposed in the literature. Unfortunately, it is difficult to assess their relative performance because they are implemented in different software tools that:

1. use different TFBS representations.

2. use different search methods.

3. are tested on different data sets in the original papers.

We compare how several scoring functions perform on both real and semi-simulated data sets in a common test environment. We also develop two new scoring functions and include them in the comparison.

## Results

### Scoring function performance

**Figure 1:**
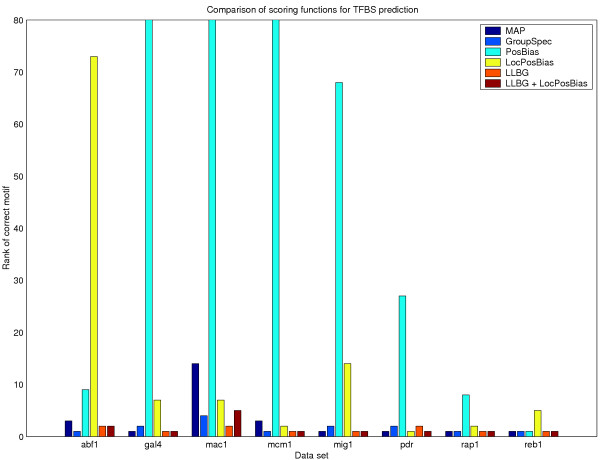
Comparison of scoring functions on eight different data sets (lower rank is better)

Six scoring functions (described in detail in the Methods section) have been evaluated in this study. The scoring functions were tested on eight different yeast data sets (Fig. 1). In order to compare the performance of the different scoring functions, the rank of the correct motif is shown. Lower rank is better, since the rank is the position of the correct motif in the list of all potential motifs, sorted according to the score from each scoring function.

MAP is performing quite well on most data sets, except for mac1. The same holds for Group Specificity. Positional Bias performs poorly for most data sets, except reb1, abf1 and rap1. Local Positional Bias is clearly better than Positional Bias, but has problems with abf1 and mig1. A closer look at the poor performance of Local Positional Bias for abf1 reveals that the positions of the correct binding sites are not clearly localized to a certain region of the promoter sequences. There is only a weak local positional bias (0.18), and many random motifs show a higher local positional bias.

LLBG performs well for all data sets and is the best scoring function in this comparison. The linear combination of LLBG and Local Positional Bias performs well in general, but has some problems with mac1, where it interestingly performs worse than LLBG alone. The reason is that Local Positional Bias performs significantly worse than LLBG for this data set. Please note that ranks are shown in the graph, so the combined score is not a linear function of the bar heights of LLBG and Local Positional Bias, but a linear function of the actual score values (data not shown) of the LLBG and Local Positional Bias.

In order to avoid overfitting, the value of the Local Positional Bias weight *a*_2 _was estimated based on all data sets but the current one. The value of *a*_2 _was around 0.5 for all data sets (the LLBG weight *a*_1 _was fixed to 1).

### Addition of noise

**Figure 2:**
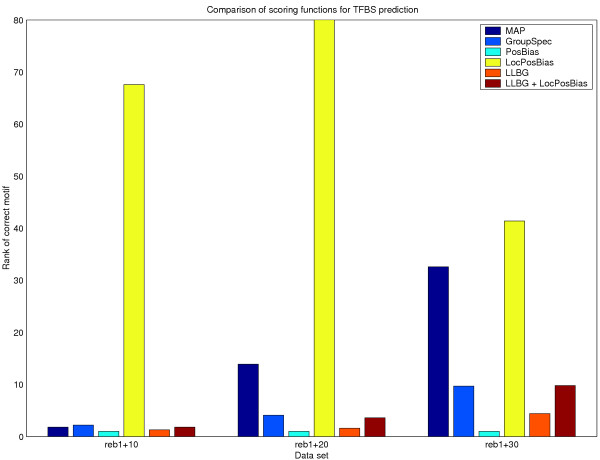
Comparison of scoring functions on the reb1 data set with different amounts of added noise. The average of ten independent runs is shown (lower average rank is better).

**Figure 3:**
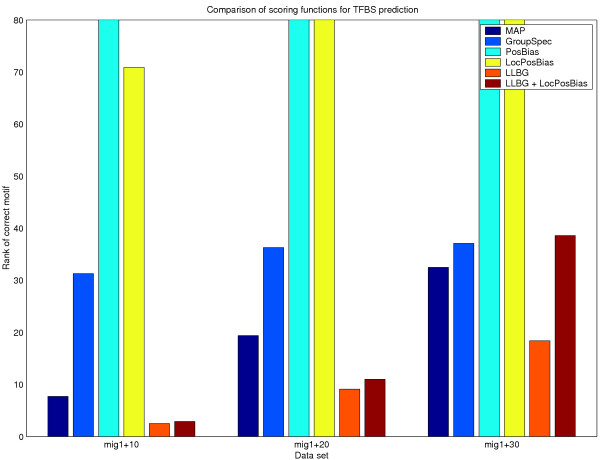
Comparison of scoring functions on the mig1 data set with different amounts of added noise. The average of ten independent runs is shown (lower average rank is better).

Generally, it is not realistic that all sequences in a data set contain the binding site of interest. Often, when the set of potentially co-regulated genes is defined by microarray experiments (gene expression profiling), there are false positives (genes where the TFBS of interest is not present) in the data set. In order to evaluate the scoring functions in the presence of this biologically relevant noise, between 10 and 30 promoter sequences were randomly selected from the genome and added to the reb1 (Fig. 2) and mig1 (Fig. 3) data sets (in the original comparison without noise, reb1 was the easiest data set to predict, and mig1 was a more difficult one, for which the best scoring functions performed about equally well). The results shown are the average of ten independent runs, with different randomly selected promoter sequences added in each run. Table 4 contains a summary of the results.

#### reb1

In the reb1 data set (Fig. 2) the MAP score performs well for reb1+10 (the original reb1 data set plus 10 randomly selected upstream sequences). However, for reb1+20 and reb1+30, performance decreases quickly. Group Specificity shows a similar trend, but is clearly better than MAP for reb1+20 and reb1+30. Interestingly, Positional Bias performs extremely well on this data set. However, because of its general bad performance (Fig. 1), we should not put too much confidence into this scoring function. Local Positional Bias performs consistently poorly on this data set. LLBG does extremely well on reb1+10 and reb1+20, and for reb1+30 it also shows a good result. The combined score performs quite well, but consistently worse than LLBG alone. The reason, as we can see, is that Local Positional Bias does not perform well on this data set.

#### mig1

The mig1 data set (Fig. 3) was more difficult. The reason seems to be that the mig1 motif shows higher variability between the different promoter sequences than does the reb1 motif. MAP performed quite poorly, especially on mig1+20 and mig1+30. Group Specificity, Positional Bias and Local Positional Bias failed already at mig1+10. LLBG performed best in every case, slightly better than the combined scoring function on mig1+10 and mig1+20, and significantly better on mig1+30.

All in all, LLBG seems to be the best scoring function in this study.

## Discussion

### Choice of search method

The iterative deterministic search method was used in this study because it has been shown [8,14] to suffer less from local optima than e.g. Gibbs sampling. However, the scoring functions tested here can score any arbitrary set of candidate words, no matter how these words are selected, so the relative performance of the different scoring functions should not depend on the choice of search method. The only interaction between the search method and the scoring function is that the search method provides the scoring function with several sets of candidate motifs to score.

### Determination of parameters

Most scoring functions have one parameter where the value is not directly determined by the data: MAP (Markov model order), Group Specificity (*s*_1_), Local Positional Bias (*L*_*w*_) and LLBG (Markov model order). In this respect, these scoring functions are similar. The exception is Positional Bias, which has two parameters (*t*_*m *_and *L*_*w*_). We also note that the cardinality of the parameters are different. For example, only a few discrete values are reasonable for the Markov model order. On the other hand, e.g. the Group Specificity *s*_1 _parameter has a larger range of possible values.

We have not tried to find the optimal parameters of each scoring function, but used the values proposed in the original papers. Since yeast is often used as a model organism for TFBS prediction studies, we assumed that the default parameters are reasonable for the yeast data sets in this study. Also, to make the comparison as fair as possible, we used the same Markov model order (3) in LLBG as was used in MAP.

### Positional bias

Because of the high variance in performance of the positional bias based scoring functions, it seems that positional bias is a feature of only a few of the data sets in this study. For others, it seems that the positions of the TFBSs do not deviate strongly from a random distribution. This has also been observed previously [6]. When a large number of long promoter sequences are searched for motifs, many candidate motifs have to be considered. If a scoring function only deviates slightly from the random distribution, many false positives will be found, which is the case of Positional Bias and Local Positional Bias.

### Advantages of LLBG

Robustness is an important property of TFBS scoring functions. The tests performed on reb1 and mig1 with added noise indicate that LLBG is quite robust against this form of biologically realistic noise, more robust than the other scoring functions in this test.

Many software tools for TFBS prediction require the user to specify the motif width *w as a *parameter. This is of course difficult when the motif is unknown and makes these tools impractical to use. The MAP scoring function is normalized by the motif width, which should make it comparable for motifs of different widths [8]. However, scaling the entropy part of MAP is problematic, as pointed out by [14]. The LLBG score does not have entropy as a part of its function, and hence should not suffer from this problem.

### Possible extensions

The LLBG score, as it is currently defined, measures the probability of a motif occurring *at least once *in the promoter sequence. It is possible to extend it to *how many times *a motif occurs in a sequence, which would increase its performance on data sets with several TFBSs per sequence. However, initial experiments have indicated that a multi-motif-per-sequence version of LLBG did not improve the results for the yeast data sets that we have been working with so far.

Even though we have here treated only the problem of finding a single motif, it is possible to generalize the LLBG scoring function to clusters of different motifs. This is of special interest in higher eukaryotes and will be subject to future work.

Currently, the LLBG is based on the discrete distance measure of number of mismatches between a candidate word and the PSFM consensus. Future research will go into using a continuous distance between a candidate word and the PSFM (not the PSFM consensus). This should make the score more robust, especially for long motifs with many uninformative positions, and it should further improve the performance of this scoring function.

We have here focused on the problem of de novo prediction of TFBSs. The related problem of TFBS recognition, where a library of known TFBSs is used to search for similar motifs in *S*, has not been considered here. Clearly, these libraries may improve TFBS prediction if the TFBS of interest happens to be similar to a TFBS already documented. Conceptually, this can easily be incorporated in the LLBG scoring function by studying the likelihood ratio between the TFBS library model and the background model.

### Limitations of current models

It should be noted that although the best scoring functions perform reasonably on these yeast data sets (with promoter regions of 1000 bp), the problem becomes much more difficult when dealing with higher eukaryotes (with promoter regions of more than 10000 bp). In that case, all of these scoring functions are likely to have problems (because of low signal-to-noise ratio), and it becomes more important to extend the models by including other sources of information, such as ChIP-chip and phylogenetic footprinting data. Since nature is able to find TFBSs with higher precision than any of the scoring functions reviewed here, we believe that the current computational models are missing some fundamental part of the transcription regulation mechanism. Future research will go into investigating the structural properties of DNA that enables transcription [15]. Interesting progress on work in this direction has been done recently for prokaryotes [16], and the related histone code has been suggested for eukaryotes [17].

## Conclusion

The time requirements of the scoring functions in this study are very different. LLBG, MAP and Local Positional Bias are relatively fast to evaluate. Group Specificity and Positional Bias are significantly more time consuming, since they require a search of a PSFM in all intergenic sequences. Since the two slower scoring functions do not perform better than three faster ones, their longer computation time does not seem to be justified.

The Positional Bias and Local Positional Bias are scoring functions that perform quite poorly for several data sets (e.g. mig1), but quite well for others (e.g. reb1 without the added noise). In other words, it seems that this feature is not relevant for some data sets (that the position of the binding site in the upstream sequence is clearly different between genes), but that it clearly matters for other data sets. This makes these scoring functions difficult to use for de novo TFBS prediction, since we cannot know beforehand whether an unknown TFBS is positionally biased.

LLBG is the scoring function that performs best in this test. The other scoring functions perform well on some data sets and poorly on others. Combining LLBG and Local Positional Bias results in a scoring function that on average performs slightly worse than LLBG alone. Since there is no clear improvement in combining the scoring functions (Fig 1, 2 and 3), the simpler solution of using only LLBG should be preferred.

A software tool using the LLBG scoring function is currently being developed.

## Methods

### TFBS representation

The PSFM representation is used for all comparisons, since it provides a good approximation of the specific protein-DNA interactions [18], and since it seems to be the representation most commonly used in the literature. The PSFM is a matrix consisting of the frequency of each nucleotide at each motif position.

### Search method

A deterministic iterative search method similar to [8,14] was used, since it thoroughly searches the promoter sequences in quite reasonable time (less than a minute for most data sets of typical size, around 20 sequences of 1000 bp each). Furthermore, it does not suffer from the problem of local minima to the same degree as Gibbs sampling, as pointed out in [8,14]. In short, it works as follows:

The first word *W*_*b *_of width *w *in *S*_1 _(position 1..*w*) is chosen as base word. A candidate set of words is collected for which the hamming distance to the base word is at most *m*, a threshold which is determined empirically as a function of *w *[14]. Each sequence *S*_*i *_may contribute zero, one or several words to the set of candidate words. A PSFM is created from all the *N*_*c *_candidate words by computing the frequency of each nucleotide at each position. This PSFM is iteratively refined by removing words until a scoring function is maximized. This is repeated for all base words *W*_*b *_in *S*_1 _(position 2..*w *+ 1, 3..*w *+ 2, ...), generating one PSFM for each *W*_*b*_. In order to improve the results, a search is done using each PSFM against *S *to define a new set of candidate words, which are again iteratively refined a maximum number of times or until convergence. For the purpose of searching for a PSFM in *S*,  pseudo-counts are used as described in [13].

The data sets in this study were known to have a motif occurrence in *S*_1_, so we only used base words from *S*_1 _for efficiency reasons, as was done in [14]. However, in general this cannot be assumed, so base words should normally be chosen from several (or all) sequences in *S*. Base words were collected from both strands.

### Scoring functions

The following scoring functions were tested: MAP [8], Group Specificity and Positional Bias [6]. We also introduced two new scoring functions: LLBG and Local Positional Bias, and included them in the comparison.

#### MAP

The MAP (maximum a posteriori probability) score is used in MDscan [8]. It is a combination of the negative entropy of the PSFM and the rareness of the PSFM according to a 3rd order Markov model estimated from all intergenic regions of a genome:



where *w *is the width of the motif, *x*_*m *_is the number of candidate words (*m*-matches) in the PSFM, *p*_*ij *_is the frequency of nucleotide *j *at position *i *of the PSFM and *p*_0_(*s*) is the probability of generating the candidate word *s *from the background model. We computed *p*_0 _using a 3rd order Markov model in the following way (for the example word ACAGT):

*p*_0_(*ACAGT*) = *p*(*ACA*)*p*(*G*|*ACA*)*p*(*T*|*CAG*)     (2)

The first part of the MAP score is the negative entropy, which is higher for PSFMs with more similar candidate words. A PSFM with identical words has maximum negative entropy (0), and a PSFM where all nucleotides are equally frequent at each position has minimum negative entropy (-2*w*). Naturally, true TFBSs are expected to be similar (and in some rare cases even identical) words.

#### Group specificity

This score is used in AlignACE [6]. It measures how well a given motif is localized to the set of input sequences *S *compared to all non-coding sequences. The rationale is that the true TFBS is a motif that is clearly more frequent in the selected promoter sequences than in all promoter sequences.

All promoter sequences in the genome are searched for the motif PSFM. The set of sequences from the top *s*_1 _hits are intersected with *S*, and the probability that these two sets would have the observed intersection or greater (Group Specificity score) is calculated:



where *T *is the total number of promoter sequences, *s*_1 _is the top number of genes (typically 100), *s*_2 _is the number of sequences in *S*, and *x *is the number of ORFs in the intersection of the two lists. This score has the advantage (compared to a *k*th-order Markov model) that it estimates how rare the motif PSFM is, not the rareness of parts of each candidate word in the PSFM.

#### Positional Bias

Like Group Specificity, this score was proposed in AlignACE [6]. It measures the concentration of motifs within a certain distance from the transcriptional start site. The rationale is that TFBSs tend to be located at the same distance from the transcriptional start site of each gene. Since the transcriptional start site is difficult to map, the translational start site is used as a reasonable approximation. The top *t*_*m *_(typically 200) PSFM hits in the genome for a given motif are found and their positions relative to the nearest ORF start are extracted. Among these, the *t *PSFM hits that are found within *L *bp upstream of some ORF are considered further. Let *m*_*w *_be the largest number of hits found in any *L*_*w *_bp window of the upstream sequences. The probability (Positional Bias) of observing *m*_*w *_or more sites out of the maximum possible *t *is determined by a binomial distribution:



where



In the original paper, *L*_*w *_= 50 and *L *= 600 were used. Because of longer upstream sequences in the data sets used in this paper (*L *= 1000), we chose a larger window (*L*_*w *_= 100).

#### Local Positional Bias

Initial tests showed that the original Positional Bias performed poorly on some data sets, so we decided to try a modified version of it. We call this new scoring function Local Positional Bias. It differs from Positional Bias in two ways:

1. The positional bias is measured in the input sequences *S *instead of the whole genome.

2. The bias of all windows is considered (using a *χ*^2^-test) instead of only the window with the largest number of sites. This makes it less sensitive to noise.

**Figure 4:**
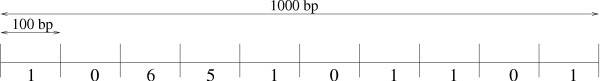
Example of Local Positional Bias calculation: A promoter sequence of 1000 bp is split into windows of 100 bp each. 16 motif occurrences are distributed over the 10 windows.

The Local Positional Bias *P*_*loc *_is defined as the probability (according to a *χ*^2^-test) that the positions of the sampled motifs were generated from a model where all positions are equally probable. For example, consider Fig. 4 with 16 motif occurrences distributed over *w *= 10 windows. The test statistic is computed as:



where *C*_*i *_is the number of occurrences in window *i *and *E*(*C*_*i*_) is the expected number of occurrences in each window, in this example 16/10 = 1.6. The *χ*^2 ^test statistic has *f *= *w *- 1 degrees of freedom, in our case *f *= 10 - 1 = 9. In our example, *χ*^2 ^= 25.25, which leads to a probability (Local Positional Bias) of 0.0027 according to the cumulative *χ*^2^-distribution.

#### LLBG

In the LLBG (least likely under the background model) score we consider the likelihood that a motif occurs at least once in *M *promoter sequences out of *N *under a background model. The idea is that the TFBS is the motif that is least likely to have been produced in these promoter sequences by the background model. The trade-off between more motif occurrences and lower probability according to the background model is treated in a probabilistic manner.

Given a set of candidate words (and a PSFM created from these words), we define the worst candidate word *W*_*w *_as the word with the largest Hamming distance *d*_*max *_to the PSFM consensus sequence *W*_*c*_. Let *p*_*eb *_be the probability that a randomly chosen word is at most *d*_*max *_from the consensus:

*p*_*eb *_= *Pr*[*d*(*W*, *W*_*c*_) ≤ *d*_*max*_]     (7)

where *d *is the Hamming distance function and *W *is a random word from the 3rd order Markov model of all intergenic sequences. In the trivial case where all candidate words are identical (*d*_*max *_= 0), there is only one word for which *d*(*W*, *W*_*c*_) ≤ *d*_*max*_. In this case, *p*_*eb *_= *p*_0_(*W*_*w*_), where *p*_0 _is computed like in equation (2). If *W*_*w *_is at distance 1 from *W*_*c*_, we have to sum the *p*_0 _of 1 + 3*w *different words. In the general case, we have to consider *n*_*w *_words:



and sum the *p*_0 _of all *n*_*w *_words to get *p*_*eb*_. However, it becomes intractable to sum over all the possibilities for many mismatches in long motifs. Instead, we use the approximation:



where *p*_*avg *_is the average *p*_0 _of the total set of words with Hamming distance at most *d*_*max *_from *W*_*c *_and  is the average *p*_0 _of the candidate words. While *p*_*avg *_is often intractable to compute,  can be computed much faster. In practice, the candidate words serve as a good representation of the total set of words of interest. Furthermore, the variance of *p*_0 _of different words within these sets is usually low, which makes  very close to *p*_*avg*_.

Having defined *p*_*eb*_, the probability of the motif occurring at least once in a promoter sequence of length *L *(assuming that the motif has equal probability to occur at all positions) is:

*p*_1*s *_= 1 - (1 - *p*_*eb*_)^*L*-*w*+1 ^    (10)

For small values of *p*_*eb*_, we can approximate equation (10) using Maclaurin polynomials:

*p*_1*s *_≈ (*L *- *w *+ 1)*p*_*eb *_    (11)

Holding the first occurrence of the motif fixed, the probability of the motif occurring in at least *M *- 1 additional promoter sequences out of the total *N *- 1 is:



which is our LLBG score. Since each occurrence is compared to the consensus, it can be argued that we should consider *M *occurrences. The reason that we consider *M *- 1 instead of *M *additional promoter sequences is that the latter introduces a bias when comparing scores from motifs of different lengths (long motifs with very few occurrences get too high scores). In the extreme case, consider a very long motif with only one occurrence: If *M *were used, this motif would get higher scores than all the biologically relevant motifs. By removing one occurrence (the most consensus like) and only considering the remaining *M *- 1 occurrences, this problem is solved.

#### LLBG + Local Positional Bias

In addition, we considered a combined scoring function of LLBG and Local Positional Bias. The correlation coefficient between these scoring functions is low (Table 1) and they are independent (within one standard deviation) according to a *χ*^2^-test (Table 2).

Generally, scores can be combined by converting them to normal deviates (using the z-transform) and summing them together. However, LLBG and Local Positional Bias deviate quite clearly from a normal distribution (data not shown), so we do not consider this option. Instead, since these scores are probabilities, we can combine them by adding their logarithms together:

*comb *= log *p*_*ms *_+ log *P*_*loc *_    (13)

However, as pointed out in [19], giving the scores equal weight often causes problems since one scoring function may be so dominating that the contribution of the other is practically ignored. Instead, we give each scoring function a unique weight:

*comb *= *a*_1 _log *p*_*ms *_+ *a*_2 _log *P*_*loc *_    (14)

In practice, we are only interested in the relative weighting between the two scores, so we fix one (*a*_1 _= 1) and find the optimal value of the other. In order to avoid overfitting, we do an *n*-fold cross validation (e.g. when combining the scores for the reb1 data set, the parameters are optimized on all data sets except reb1).

As was shown in the results section, this combined scoring function did not perform better than LLBG alone.

### Data sets

The scoring functions were tested on data sets from [14]. These data sets consist of the promoter sequences (1000 bp upstream regions) of genes regulated by a certain transcription factor in yeast (Table 3). The 1000 bp upstream regions were used even in those cases where it overlaps another ORF. Each promoter sequence has at least one (putative or biologically verified) binding site of the transcription factor of interest. Most data sets are compiled from different molecular biology studies, where each binding site is biologically verified (Table 3). It is possible (and likely) that the promoter sequences also contain some binding sites of other transcription factors, but this is not considered here, as we do not have sufficient information on this. The width of the correct motif was given to the search algorithm. This is not needed by the search algorithm or scoring functions used in this comparison, but it facilitates the definition of what should be regarded as the correct motif. This simplification has also been used in comparisons of TFBS prediction software [14].

Each of these data sets were analyzed using each of the scoring functions. All PSFMs generated by the search method were scored using each of the scoring functions, and the complete list of PSFMs was sorted according to the score (highest first). If the consensus motifs of several PSFMs were identical, the lower scoring duplicate PSFMs were removed from the list. The rank (position in the sorted list) of the correct motif was compared between the different scoring functions for each data set.

As 'correct motif' we considered the known TFBS consensus (Table 3), either exact or shifted one position. Long motifs (i.e. at least ten informative positions) are allowed to have one mismatch or be shifted up to two positions. The reason for this relaxed definition is that the reported TFBS consensus motif for some data sets is not absolutely correct. For example, the given GAL4 binding site differs from recent findings from ChIP-chip experiments, as pointed out by [14]. Also, sometimes the first or last positions of the consensus are about as significant as the positions outside of these two (and hence not contained in the consensus sequence). This relaxed definition makes the comparison less sensitive to random fluctuations.

## Authors' contributions

GG introduced the LLBG scoring function. PvR introduced statistical ideas and performed statistical analysis. MF further developed the LLBG scoring function, implemented the scoring functions and search method, and drafted the manuscript. All authors read and approved the final manuscript.

## Figures and Tables

**Table 1:** Correlation coefficients between scores

	GroupSpec	PosBias	LocPosBias	LLBG
MAP	0.36	0.06	0.02	0.43
GroupSpec		-0.19	-0.02	0.26
PosBias			0.01	0.02
LocPosBias				*0.04*

**Table 2:** *χ*^2 ^independence test (standard deviations)

	GroupSpec	PosBias	LocPosBias	LLBG
MAP	8.48	0.66	1.25	9.00
GroupSpec		3.86	1.09	5.62
PosBias			0.47	1.27
LocPosBias				*0.99*

**Table 3:** Data sets of promoter sequences of genes regulated by different transcription factors. By 'molecular biology approaches' we mean methods like DNAse footprinting and methylation interference. 'AlignACE' stands for functional group data from the AlignACE web server. '#seqs' stands for number of promoter sequences in the data set.

TF	TFBS consensus motif	#seqs	source and type of evidence
abf1	CGTNNNNNNTGA	20	molecular biology approaches [20]
gal4	CGGNNNNNNNNNNNCCG	10	molecular biology approaches [20] and AlignACE [6]
mac1	TTTGCTCA	6	microarray [21]
mcm1	TTTCCCAAANNGGAAA	24	molecular biology approaches [20]
mig1	AAAAATCTGGGG	11	molecular biology approaches [22]
pdr	TCCGCGGA	11	AlignACE [6]
rap1	TACACCCATACATT	44	molecular biology approaches [23] [24]
reb1	TTACCCG	13	molecular biology approaches [20]

**Table 4:** Schematic interpretation of the results for the reb1 and mig1 data sets with added noise, good: top 3, ok: top 10, bad: worse than top 10

	MAP	GroupSpec	PosBias	LocPosBias	LLBG	LLBG LocPosBias
reb1	good	good	good	ok	good	good
reb1+10	good	good	good	bad	good	good
reb1+20	bad	ok	good	bad	good	ok
reb1+30	bad	ok	good	bad	ok	ok

mig1	good	good	bad	bad	good	good
mig1+10	ok	bad	bad	bad	good	ok
mig1+20	bad	bad	bad	bad	ok	bad
mig1+30	bad	bad	bad	bad	bad	bad
